# Mandible Thickness in the Pediatric Population: A Pilot Normative Anthropometric Estimation Using CT Images

**DOI:** 10.7759/cureus.91507

**Published:** 2025-09-02

**Authors:** Anuvindha JS, Prudhvinath Reddy, Shikha Yadav, Rajeev Aravindakshan

**Affiliations:** 1 Department of Dentistry (Oral and Maxillofacial Surgery), All India Institute of Medical Sciences Mangalagiri, Mangalagiri, IND; 2 Department of Radiology, All India Institute of Medical Sciences Mangalagiri, Mangalagiri, IND; 3 Department of Community and Family Medicine, All India Institute of Medical Sciences Mangalagiri, Mangalagiri, IND

**Keywords:** anthropometric, children, jaw, mandible, paediatric

## Abstract

Introduction

The mandible's morphology in Indian pediatric patients, shaped by genetic, environmental, and functional factors, is critical for diagnosing anomalies and planning surgical interventions, especially implant placement. However, there is scarce morphometric data on mandibular bone thickness in Indian children, coupled with the reliance on adult-focused studies and outdated imaging, which underscores the need for precise evaluation. This study aimed to address these gaps related to the topic

Methodology

We sought to assess normative anthropometric data from CT scans of 29 patients aged 2-16 years, sourced from a tertiary care hospital’s database. Using a retrospective approach, the mandible was classified into anterior and posterior segments, with full and cortical thicknesses measured at nine key points.

Results

Our findings revealed no significant bilateral differences. Maximum full and cortical thicknesses were observed at point 7 (upper one-third, distal-most body region). At the same time, minimum values were at point 3 (lower one-third, symphysis-parasymphysis) for full thickness and point 1 (upper one-third, symphysis-parasymphysis) for cortical thickness.

Conclusions

This study provides normative mandibular thickness data for Indian children, offering a foundation for global mandibular assessment across ages and genders, thereby enhancing the safety and efficacy of dental and surgical interventions.

## Introduction

Craniofacial morphology involves a complex interplay of genetic, environmental, and functional factors, leading to significant phenotypic variations. The craniofacial structures, which include oral soft and hard tissues, are notably influenced by masticatory forces [1,2]. The evolution of the mandible in hominins - particularly australopiths and species of the genus Homo - has occurred at a faster rate compared to other primate clades. This rapid evolution is linked to shared traits related to habitat preferences, body size, sexual dimorphism, diet, and food processing behaviors. Hominins display distinctive mandibular characteristics, including short, deep mandibles and smaller incisors and canines, which are believed to have evolved in response to tougher diets and changing environments [3]. Factors, such as the loss of the honing complex and canine reduction, have contributed to the high evolutionary rates observed.

During development, the mandible grows vertically (the ramus) more than horizontally (the body), with most growth occurring in the first six months of life. Models by Schipper et al. provide insights into normal mandibular development during the first two years, which is crucial for diagnosing developmental disorders such as micrognathia and macrognathia [4]. The morphology and bone quantity in the mandible are vital for surgical planning, especially for implant placement. Cortical bone thickness is significant for initial stabilization, often more than the implant length itself. Consideration of tooth buds during surgical procedures is crucial, as damage to them can hinder the eruption of permanent teeth [5]. Although miniscrews are commonly used for orthodontic anchorage, data on their use in mixed dentition are limited [6,7]. Accurate knowledge of bone thickness is essential when selecting screw lengths for open reduction and internal fixation (ORIF) [8]. Traditional methods for mandibular evaluation, such as cadaveric studies and 2D imaging, have certain limitations and are prone to error [2]. CT provides better insights.

There is a significant gap in morphometric data on mandibular bone thickness in Indian pediatric populations. Existing studies focus primarily on adults, mostly caucasians, or use limited imaging techniques. The objectives of this study were to evaluate the thickness of the buccal and labial cortical plates and to assess the total mandibular thickness (labiolingual and buccolingual) in the pediatric population. Additionally, it also sought to determine the presence of nerve and tooth buds or tooth structures in the areas of interest, thereby providing information on safe zones for implant placement.

## Materials and methods

A retrospective, record-based data analysis was conducted by utilizing CT scan images of the head and neck region, specifically focusing on the mandible. The study analyzed existing imaging database archives of the head and neck regions, captured with a Siemens Somatom Drive Dual-Source 256-Slice CT Scanner (Siemens Healthineers USA, Malvern, PA) in the Department of Radiodiagnosis at a tertiary care hospital. By leveraging this institutional database, the research aimed to evaluate the anatomical features of the mandible while avoiding unnecessary radiation exposure to the subjects. The minimum sample size to estimate normative values based on a single group mean with a required absolute precision of 0.15, given a standard deviation of 0.41 at a 95% confidence level, was calculated as 29 [7].

This investigation encompassed a cohort of pediatric patients aged 2-16 years. To assure precise developmental measurements, subjects were excluded based on criteria such as suboptimal scan quality, the presence of mandibular pathologies or fractures, syndromic conditions, pronounced facial or dental asymmetries, systemic diseases, extensive medical or dental histories, and any indications of vertical or horizontal periodontal bone loss. Images were analyzed using Singo Via software and oriented in sagittal, axial, and coronal planes. All measurements were performed by a single trained examiner (Senior Resident AJS) under the supervision of consultants from Oral and Maxillofacial Surgery (SY) and Radiology (PR). The examiner was trained on the measurement protocol and evaluated for consistency before commencing the study. The mandible was systematically segmented (anterior and posterior) into vertical and horizontal sections to facilitate thorough data collection. Multiple lines delineated specific anatomical landmarks to standardize measurement locations across the sample.

**Figure 1:**
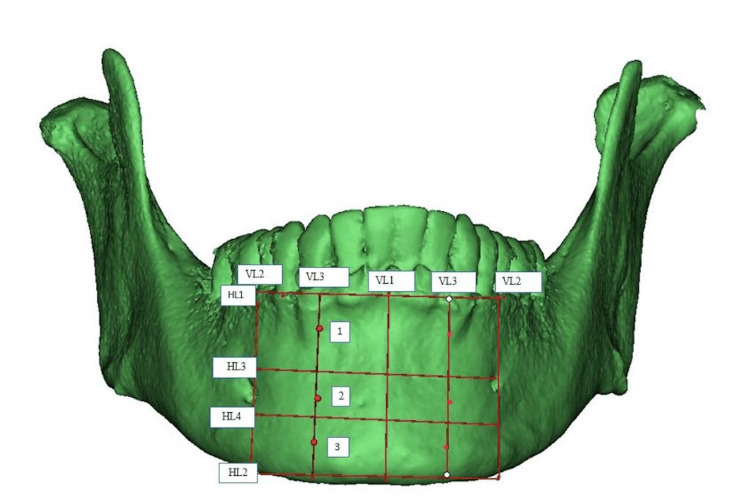
Representative image of the segmentation of the mandible - 2

The anterior segment was defined between two vertical lines (VL2) drawn through the mental foramen on both sides, as shown in Figure 1. This segment was further divided into vertical and horizontal sections using additional vertical lines (VL1 and VL3) and horizontal lines (HL1, HL2, HL3, and HL4). Vertically, it was divided into four sections: VL1, drawn from the midline, divided the anterior segment into two vertical halves, and VL3, drawn equidistant from VL1 and VL2 on both sides, further subdivided each half. Horizontally, it was divided into three sections: HL1 along the cemento-enamel junction (CEJ), HL2 along the lower border, and HL3, which divided the segment into equal halves. HL4, drawn equidistant from HL3 and HL2, further divided the lower horizontal section. Points 1, 2, and 3 were marked along VL3 at the mid-distance between HL1-HL3, HL3-HL4, and HL4-HL2, on both sides, respectively

**Figure 2:**
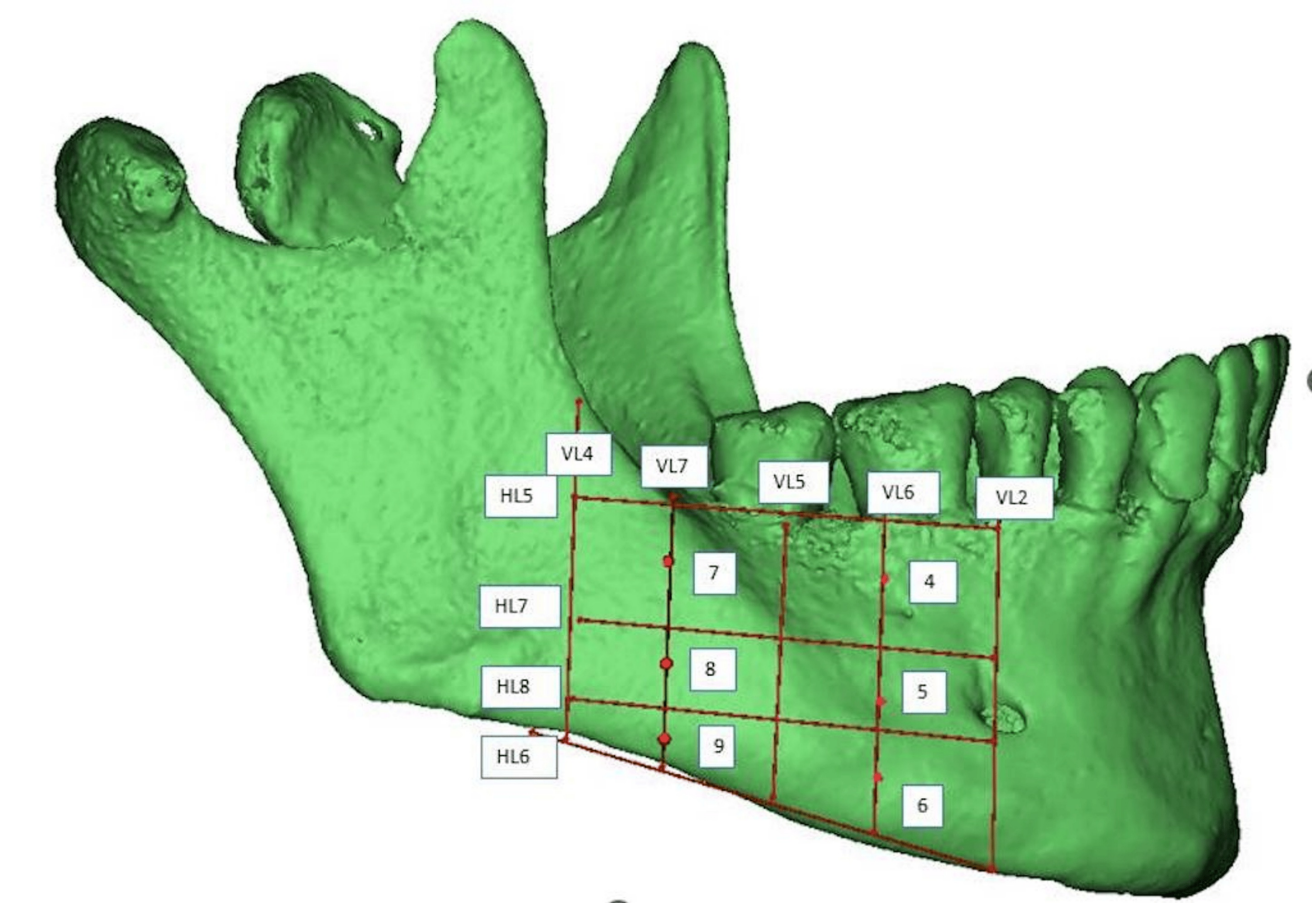
Representative image of the segmentation of the mandible - 2

As shown in Figure 2, each posterior segment was also divided into vertical and horizontal sections, using vertical lines (VL2, VL4, VL5, VL6, VL7) and horizontal lines (HL5, HL6, HL7, HL8). Vertically, it was divided into four sections: VL2 through the mental foramen, VL4 along the anterior border of the ramus, VL5 dividing the segment into equal halves, and VL6 and VL7 dividing each half further. Horizontally, it was defined by the region between HL5 and HL6, along the CEJ and the lower border of the jaw. HL7 divided this segment into equal halves, and HL8 further divided the lower half equally. Points 4, 5, and 6 were marked along VL6 at the midpoint between HL5 and HL7, HL7 and HL8, and HL8 and HL6, respectively. Similarly, points 7, 8, and 9 were marked along VL7 at the midpoint between HL5 and HL7, HL7 and HL8, and HL8 and HL6.

**Figure 3:**
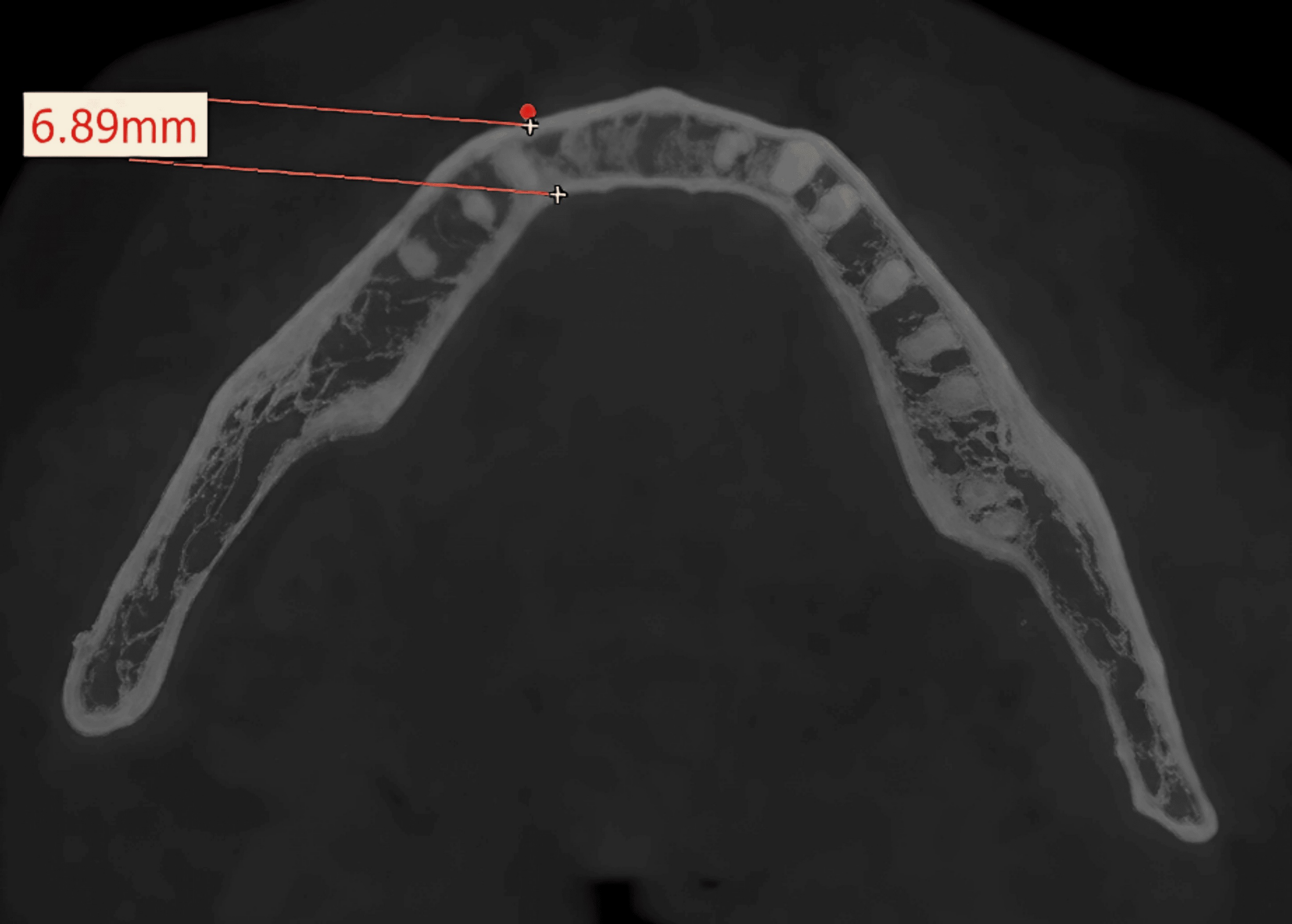
Representative image of the manner of measurement of the full thickness of the mandible (labiolingual)

**Figure 4:**
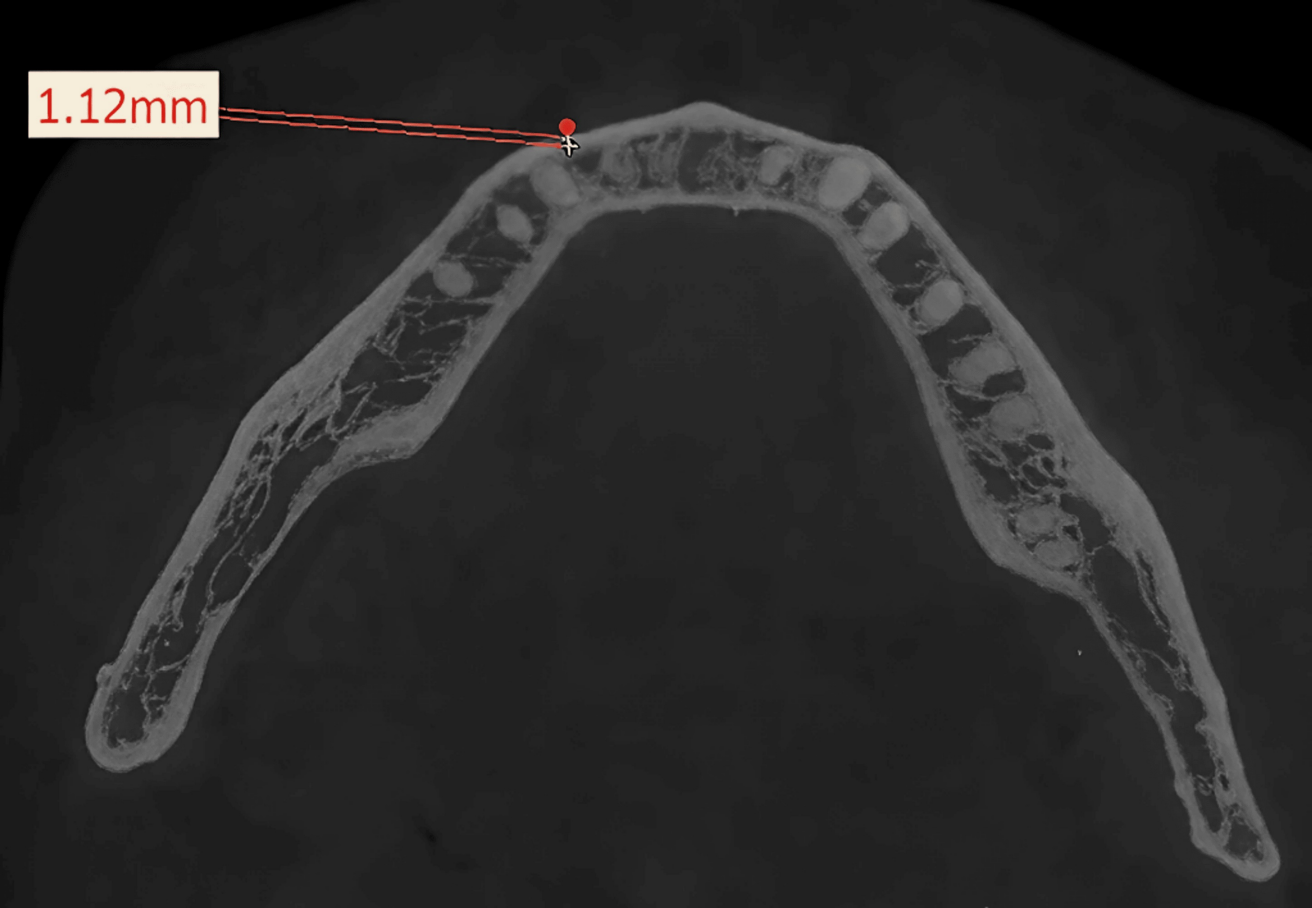
Representative image of the manner of measurement of the thickness of the buccal cortex

Measurements at designated center points, labelled from 1 to 9, were accurately recorded on both the right and left sides, and the full thickness of the mandible (buccal cortex to lingual cortex; labial cortex to lingual cortex) and labial/buccal cortical thickness were measured at the marked points (Figures 3, 4). Any presence of a tooth bud/tooth structure or nerve canal was documented at these locations during the evaluation. 

Data were entered into a Microsoft Excel spreadsheet, and statistical analyses were performed using SPSS Statistics, version 17.0 (IBM Corp., Armonk, NY), EpiInfo, and R software. Measurements at points 1-9 were expressed as means and standard errors in millimetres and statistically analyzed using the Wilcoxon Signed-Rank and Mann-Whitney U tests across different levels. P-values less than 0.05 were considered statistically significant.

To ensure measurement reliability and assess intra-observer reliability, each scan was independently evaluated twice by the same assessor, with a two-week interval between sessions. The repeated measurements demonstrated high correlations (Pearson’s r > 0.90, p < 0.001 for all assessed points), indicating strong agreement and confirming excellent intra-observer reliability. Additionally, the differences between the two sets of measurements were tested using paired t-tests, which revealed no statistically significant differences (p > 0.05 for all points). These analyses confirm both the calibration and reliability of the measurement technique used in the present study. The mean of these two evaluations was used as the definitive measurement for the study.

## Results

The total number of subjects whose CT scans were assessed was 29: 14 males and 15 females. The mean age was 10.31 ± 3.77 years (range: 4-16 years). Table 1 shows the mean and standard deviation (SD) values of full-thickness (labiolingual/buccolingual) as well as cortical (buccal/labial) mandibular thickness at all the assessed points.

**Table 1:** Comparison between mean thickness on the right and left side ^*^P-value byWilcoxon signed-rank test (significant at p<0.05). Full refers to the distance from buccal to lingual cortex; labial to lingual cortex at the point mentioned; C is the thickness of the buccal or labial cortex at the point of measurement SD: standard deviation

Points	Right mean (mm)	SD	Left mean (mm)	SD	P-value^*^
1 full	11.31	1.86	11.17	1.73	0.689
1 c	1.51	0.35	1.50	0.48	0.829
2 full	10.46	1.41	10.20	1.56	0.294
2 c	1.57	0.37	1.56	0.44	0.974
3 full	9.28	1.37	9.02	1.27	0.531
3 c	1.72	0.31	1.76	0.39	0.546
4 full	12.24	2.14	12.10	2.18	0.857
4 c	2.19	0.68	2.09	0.49	0.313
5 full	12.22	1.79	12.01	2.08	0.927
5 c	2.19	0.52	2.11	0.57	0.194
6 full	10.72	1.29	10.68	1.33	0.733
6 c	2.16	0.54	2.14	0.56	0.946
7 full	14.45	2.30	14.48	2.47	0.633
7 c	2.61	0.76	2.61	0.81	0.873
8 full	13.20	1.92	12.76	1.86	0.101
8 c	2.35	0.47	2.37	0.61	0.855
9 full	10.70	1.54	10.60	1.30	0.991
9 c	2.48	0.61	2.39	0.64	0.182

The Wilcoxon signed-rank test revealed no significant bilateral differences in mandibular thickness at any of the nine points of assessment in all the subjects. The maximum full thickness of the mandible (buccal cortex to lingual cortex; labial cortex to lingual cortex) was consistently located at point 7, showing no significant side difference (right: 7 full, 14.45 ± 2.30 mm; left: 7 full, 14.48 ± 2.47 mm; p = 0.63). Similarly, the minimum full thickness observed was at point 3 (right: 3 full, 9.28 ± 1.37 mm; left: 3 full, 9.02 ± 1.27 mm; p = 0.53). Analysis of cortical thickness (buccal/labial) also indicated no significant bilateral difference, with the maximum at point 7 (right 7c 2.61 ± 0.77 mm; left 7c 2.61 ± 0.81 mm; p = 0.87) and the minimum at point 1 (right 1c 1.51 ± 0.35 mm; left 1c 1.50 ± 0.48 mm; p = 0.82). 

**Figure 5:**
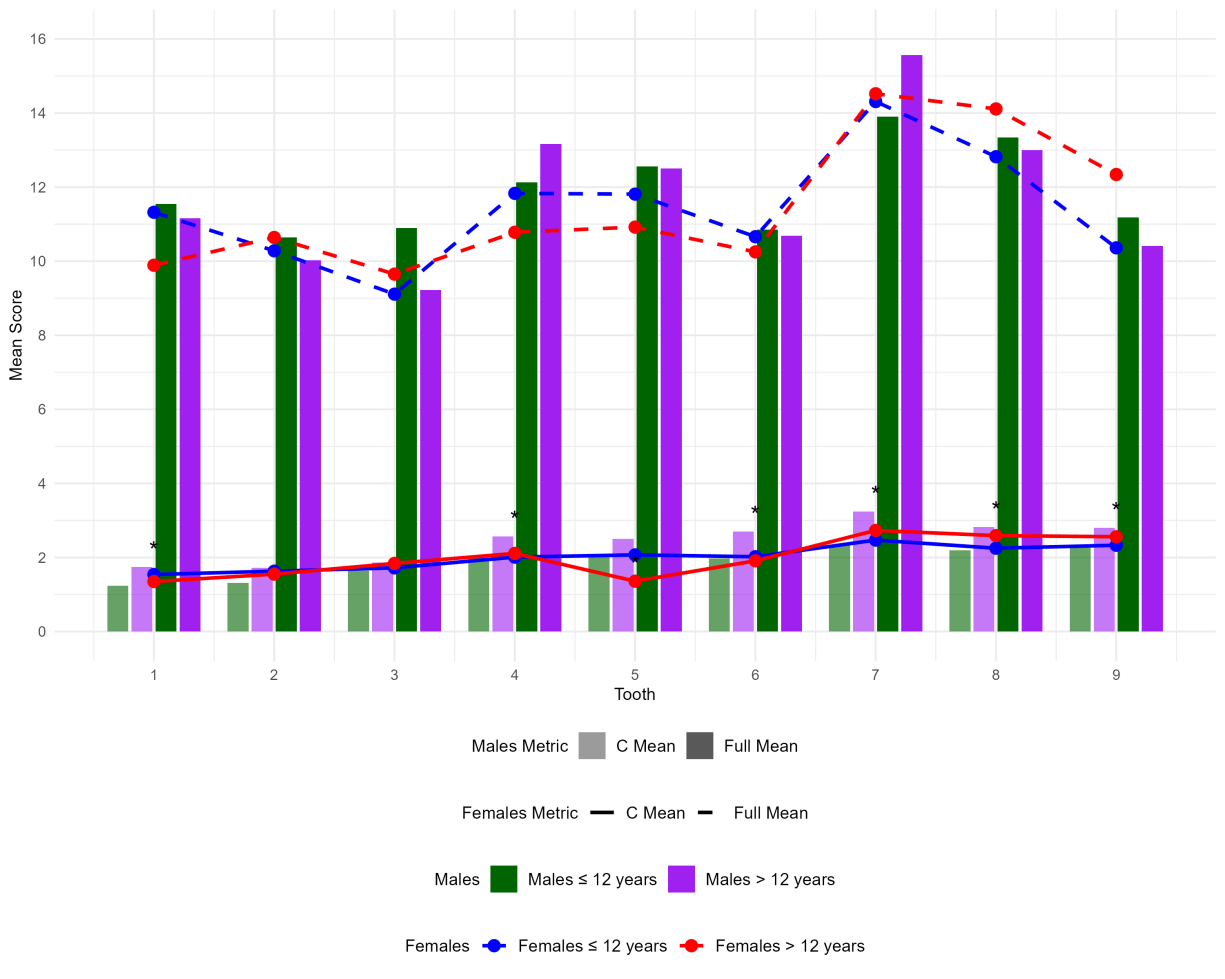
Age- and gender-based subgroup analyses

Age (2-12 years and 13-16 years) and gender subgroup analyses showed no significant variance in mandibular full thickness across the groups. However, there were substantial differences in cortical thickness between the two age groups (2-12 years and 13-16 years) at points 4 and 6-9 (Figure 5).

No significant differences were observed between females and males at any point, as shown in Table 2.

**Table 2:** Comparison between females and males at all 9 points ^*^P-values by Mann-Whitney U test (significant at p < 0.05). Full refers to the distance from buccal to lingual cortex; labial to lingual cortex at the point mentioned; C is the thickness of the buccal or labial cortex at the point of measurement SD: standard deviation

Points	Females, mean (mm)	SD	Males, mean (mm)	SD	P-value^*^
1 full	11.13	1.38	11.35	2.12	0.570
1 c	1.52	0.27	1.49	0.46	0.965
2 full	10.33	1.23	10.33	1.57	0.949
2 c	1.61	0.30	1.51	0.44	0.616
3 full	9.19	1.22	9.11	1.10	0.663
3 c	1.74	0.27	1.75	0.34	0.513
4 full	11.69	1.61	12.65	2.22	0.138
4 c	2.02	0.48	2.28	0.57	0.198
5 full	11.69	1.40	12.53	1.80	0.163
5 c	1.97	0.48	2.27	0.52	0.127
6 full	10.60	1.14	10.77	1.01	0.861
6 c	2.00	0.38	2.34	0.61	0.106
7 full	14.34	2.11	14.74	2.36	0.458
7 c	2.50	0.67	2.80	0.86	0.383
8 full	12.99	1.73	13.17	1.72	0.827
8 c	2.30	0.59	2.51	0.50	0.275
9 full	10.63	1.59	10.80	0.89	0.965
9 c	2.36	0.62	2.63	0.64	0.239

**Figure 6:**
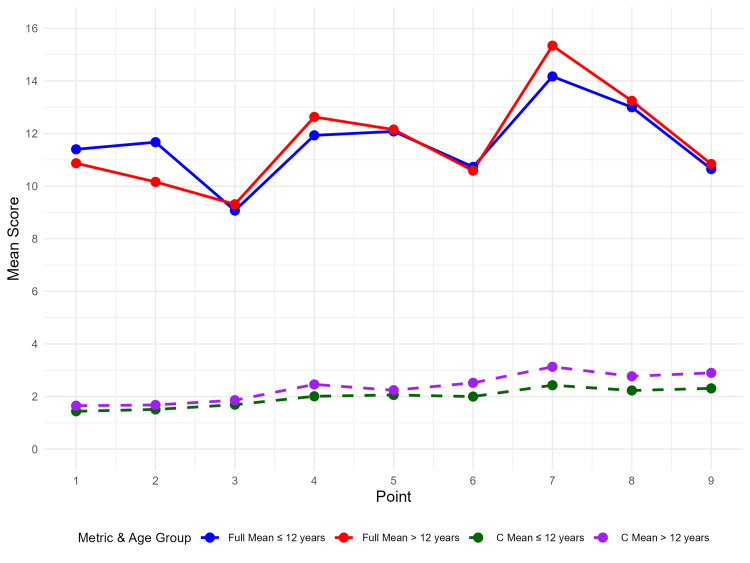
Subgroup analysis between the males aged 2-12 years and those above 12 years of age

There were no differences in thickness values among females across age groups. Males, however, exhibited differences in cortical thickness at points 6-9 (Figure 6).

Table 3 lists the findings regarding the different structures (tooth buds, tooth roots, and nerve canals) present at all assessment points. It has been tabulated on both the right and left sides, along with males and females of different age groups, such as 2-12 years and 13-16 years.

**Table 3:** Structures (tooth/nerve canal) present at points of assessment in mandibles under assessment Points 1-9; F: full thickness of mandible; C: cortical bone thickness; t: tooth present at the point of assessment; canal: nerve canal was present at the point of assessment

Right									Left							
Gender	Female				Male					Female				Male		
	t		Canal		t		Canal		t		Canal		t		Canal	
Age group, years	2-12	13-16	2-12	13-16	2-12	13-16	2-12	13-16	2-12	13-16	2-12	13-16	2-12	13-16	2-12	13-16
Structure 1F	13	2	-	-	7	7	-	-	13	1	-	-	7	7	-	-
Structure 1C	13	2	-	-	7	7	-	-	13	1	-	-	7	7	-	-
Structure 2F	5	1	-	-	3	1	-	-	6	-	-	-	3	2	-	-
Structure 2C	5	1	-	-	3	1	-	-	6	-	-	-	3	2	-	-
Structure 3F	1	-	-	-	1	-	-	-	2	-	-	-	1	-	-	-
Structure 3C	1	-	-	-	1	-	-	-	3	-	-	-	1	-	-	-
Structure 4F	13	2	-	-	7	7	-	-	12	1	-	-	5	7	-	-
Structure 4c	13	2	-	-	7	6	-	-	11	1	-	-	6	7	-	-
Structure 5F	5	-	-	-	3	1	-	-	6	-	-	-	2	-	-	-
Structure 5C	6	-	-	-	3	-	-	-	6	-	-	-	2	-	-	-
Structure 6F	1	-	1	-	2	-	-	-	2	-	-	-	1	-	-	-
Structure 6c	1	-	-	-	2	-	-	-	2	-	-	-	1	-	-	-
Structure 7F	12	2	-	-	6	6	-	-	13	1	-	-	7	7	-	-
Structure 7c	12	2	-	-	6	6	-	-	13	1	-	-	7	7	-	-
Structure 8F	5	-	-	1	2	-	-	1	3	-	-	-	1	-	1	2
Structure 8c	5	-	1	-	1	-	-	-	3	-	-	-	1	-	1	-
Structure 9F	1	-	-	-	1	-	-	-	1	-	-	-	1	-	-	-
Structure 9c	1	-	-	-	1	-	-	-	3	-	-	-	1	-	-	-

## Discussion

Human mandibular anthropometry is essential for pre-surgical treatment planning in oral and maxillofacial surgery and dental implantology. Accurate assessment of mandibular cortical bone dimensions, the mental foramen, and the positioning of the inferior alveolar nerve canal is critical, as the mandible undergoes significant variations due to ethnicity, gender, and age-related physiological changes [8]. The pediatric mandible is dynamic, with anatomy changing considerably through childhood and adolescence due to growth and functional adaptations [9]. Age-related variations in cortical bone thickness relate to increasing bite forces and masticatory muscle activity. Bone remodeling, driven by biomechanical forces and growth centers, modifies the distribution of cortical bone and the density of cancellous bone [7].

Knowledge of normal mandibular development helps identify abnormalities, although studies documenting early mandibular growth are limited. Neonatal micrognathia is common, occurring in roughly one in 500-1,600 births [4]. By the ages of 10-12 years, most mandibular growth relevant to surgery is complete, and the mental foramen moves posteriorly and vertically, generally beneath the premolars by age six. Cortical bone thickness increases significantly as dentition progresses from mixed to permanent stages [10]. Mandibular fractures constitute about one-third of pediatric facial trauma. Displaced fractures typically require ORIF, which helps overcome the challenges posed by primary and mixed dentitions. ORIF promotes rapid healing and avoids prolonged intermaxillary fixation (IMF) [11]. 

Despite the individual studies on cortical plate thickness in the literature, unambiguous conclusions about correlations should be taken with caution due to the small number and clinical heterogeneity of the included studies. One key point that needs to be addressed before discussing the present study's findings and their relationship to existing published literature is that the methodology used in the published research differs significantly from that of the present study. Establishing a foolproof association and dissociation is difficult at this point. The present study can be roughly compared to other studies. The same holds for the interrelationship between the published data. The variations in population, by ethnicity, age, gender, fresh or cadaveric status, region of assessment, method of evaluation, and others, differ significantly. While acknowledging the heterogeneity, the results should be interpreted with an open mind.

The mean age in this study was 10.31 ± 3.77 years (range: 4-16 years), and no significant difference was found between the cortical and buccolingual thickness measurements on either side. The same has been proven in earlier studies by Schwartz-Dabney and Dechow [12]. In the present study, the minimum full thickness of the mandible was at the symphysis and parasymphysis region, in the lower one-third region, which increased towards the body, and the maximum was in the upper one-third at the end of the body. Similarly, the thickness of the cortical plate was also found to be minimum in the symphysis-parasymphysis region. Still, in the upper one-third, the maximum was in the same area as the full mandibular thickness. 

Bhattad et al. [7] studied 15 Indian children aged 8-10, measuring cortical bone thickness at 6 and 8 mm from the CEJ. The results were consistent with the present study. At 6 mm, the thickness measures on the right and left sides were 2.19 mm ± 0.68 and 2.0 mm ± 0.49, respectively, and 2.19 mm ± 0.52 and 2.11 mm ± 0.57 at 8 mm. Our study included more subjects and better imaging [7]. Yasa et al. [13] measured mandibular cortical bone thickness according to BMI in 100 adolescent individuals (33 male and 67 female). They found that those who were obese or overweight had thicker mandibular cortices, which can complicate orthodontic treatments [13]. Gaffuri et al. [1] evaluated the correlation between alveolar bone thickness and facial divergence in young adults, studying 30 patients (mean age, 16 ± 2 years) with a Class I malocclusion. They found that hyperdivergent subjects had thinner alveolar bone and suggested that clinicians should be cautious about movements to avoid fenestration and dehiscence [1].

Kotsanti et al. [10] analyzed 660 panoramic radiographs of children between six and 18 years of age. They found a correlation between bone thickness and dentition type, with normal bone prevalent in children with mixed dentition. Bone quality and quantity vary significantly between different genders and age groups [10]. Hutchinson et al. studied 45 mandibles across three age groups: prenatal (30 gestational weeks to birth; n = 15); early postnatal (birth to 12 months; n = 18); and late postnatal (1-5 years; n = 12) and found that changes in bone mineral density relate to dental development and biomechanical loading [14]. Sullivan et al. focused on 242 healthy children aged 0-47 months, finding a strong correlation between size and age, with minimal shape differences by sex [15]. Vasil’ev et al. [16] reported an average cortical plate thickness of 1.57 mm at 6 years, decreasing to 1.52 mm at 7 years. This is consistent with present findings, which show a proportional decrease in thickness from the cervical region to the apical projection [16].

All the studies mentioned above indicate that in younger age groups, specifically before puberty and the onset of hormonal changes, gender does not significantly influence differences in cortical bone thickness. This finding is consistent with the results of this study. Grover and Gupta [8] utilized DentaScan on 100 individuals aged 21-50, assessing areas including the symphysis and body region. They found thicker cortices and wider mandibles in males, while younger females had thicker symphyses. Older subjects showed thicker upper third cortices and wider anterior mandibles. The presented study revealed significant differences in cortical thickness between age groups (2-12 and 13-16) at points 4 and 6-9, indicating growth-related changes in the mandibular body. However, direct comparisons with Grover and Gupta's findings are limited due to the differences in the populations [8].

Heimes et al. [17] conducted a systematic review of buccal bone thickness, including 45 studies, with only one from India. This highlights the scarcity of research on Indian morphometric data and the need for broader investigations across demographics. They reported an average buccal cortical thickness in the mandible of 0.95 ± 0.58 mm anteriorly and 1.20 ± 0.96 mm posteriorly. In a related study [17], Katranji et al. [18] evaluated 28 cadaveric heads (68% male, with an average age of 73.1 years). They found buccal cortical thickness in the edentulous mandible ranged from 1.36 to 2.06 mm, while in the dentate mandible, it ranged from 0.99 to 1.98 mm and 1.24 to 2.51 mm, respectively. Despite differences in study populations, both studies observed that the thinnest areas of buccal cortical thickness were in the lower anterior region, while the thickest areas were in the upper posterior region [18].

Twenty cadavers of Indian descent (19 men and one woman), aged 50 to 76 years, provided 20 mandibles, which were assessed by Ayyapan [19]. They found that the mean labiolingual thickness ranged from 9.23 to 11.80 mm, with a minimal value of 5.542 mm in the midline region. No significant difference was noted between dentate and edentulous specimens [19]. Al-Jandan et al. found in their study of adult mandibles that the use of 3 mm screws carries the least risk of injury to the tooth root and nerve during the fixation of fractures between the canine and first molar, while 4 mm screws can be safely used in the region of the second molar, but further studies are needed to assess the stability of fixation with 3-4 mm screws [20]. There are no studies discussing the ideal screw length in pediatric poultaion in the multiple mandibular regions.

Most studies have not discussed the presence of structures, such as nerves, tooth roots, and tooth buds, during their research evaluations. However, they do emphasize the importance of considering these structures during the planning of future studies and treatment planning. Table 3 lists the presence of these structures during the evaluation of the 29 CT scans. In the present study, it was found that the 2-12-year age group had more structures, which is justified by the fact that it is a deciduous and mixed dentition age group. It is essential that, during treatment planning, not only for the pediatric population but also for adults, a thorough assessment of the presence of these structures should be done.

However, a limitation of this study is its small sample size and the wide age range of participants. Especially with children, as there is a significant change in morphology until growth completion, a well-subgrouped sample needs to be taken, considering the influence of hormones on growth and morphological changes, which necessitates pre- and post-puberty subgrouping in males and females. As this is a pilot study, it serves as a framework for future research in this area.

Despite these limitations, the present pilot study provides valuable baseline normative data on mandibular thickness in the Indian pediatric population. Such information is clinically relevant for oral and maxillofacial surgeons, orthodontists, and implantologists when planning procedures such as miniscrew placement, fracture fixation, and implant therapy in growing children. Knowledge of regional variations in cortical and full mandibular thickness, along with the proximity of tooth buds and neurovascular structures, can help improve the safety of surgical interventions and reduce the risk of iatrogenic injury. Moreover, understanding growth-related changes in mandibular morphology can assist clinicians in anticipating anatomical variations across age groups, thereby refining treatment planning in pediatric and adolescent patients.

## Conclusions

The current study concludes that the thickness of the cortical plates and the overall thickness of the mandible change with growth and age. The thickness is greater in the posterior region, as it bears more load. A significant change has been observed in both the male groups, those above and below 12 years of age, suggesting that hormonal factors may play a role. Therefore, future studies should investigate factors such as gender, age, BMI, and facial growth patterns to establish a reliable database.
